# Comparing the Polyoxometalate-Catalyzed Oxidation of C5-Containing Biomass to Formic Acid in a Taylor-Flow Microreactor and a Stirred-Tank Reactor

**DOI:** 10.1021/acssuschemeng.5c03392

**Published:** 2025-07-24

**Authors:** Jan-Dominik H. Krueger, Lukas Popp, Markus Schörner, Hans Lorenz Grau, Patrick Schühle, Jakob Albert

**Affiliations:** † Institute of Technical and Macromolecular Chemistry, 14915 Universität Hamburg, Bundesstrasse 45, 20146 Hamburg, Germany; ‡ Institute of Chemical Reaction Engineering, 9171 Friedrich-Alexander-Universität Erlangen-Nürnberg, Egerlandstr. 3, 91058 Erlangen, Germany; § Forschungszentrum Jülich, Helmholtz Institute Erlangen Nürnberg for Renewable Energy, 91058 Erlangen, Germany

**Keywords:** biomass oxidation, polyoxometalate, microreactor, Taylor-flow regime, gas−liquid mass transfer, kinetics, reactor comparison

## Abstract

Biomass valorization using polyoxometalate-based (POM) catalysts is a promising strategy for green and sustainable chemistry. In the modified OxFA process, various biogenic substrates can be selectively oxidized to the sustainable hydrogen carrier formic acid using the H_8_PV_5_Mo_7_O_40_ (HPA-5) POM catalyst in an aqueous-methanolic solution with molecular oxygen or compressed air as an oxidant. A current challenge of the mainly used stirred-tank reactors is the mass transfer limitation with respect to the effective dissolution of oxygen in the reaction media. In order to improve gas–liquid mass transfer, alternative reactor concepts are needed. Herein, we demonstrate the selective catalytic oxidation of the C5 model sugar xylose as well as the commercial C5-hydrolysate Renmatix from the Plantrose process to formic acid in a continuous Taylor-flow microreactor, allowing for effective mixing in combination with intrinsic safety and drastically reduced reaction times. This paves the way for a more efficient biomass valorization strategy with respect to industrial implementation.

## Graphical abstract

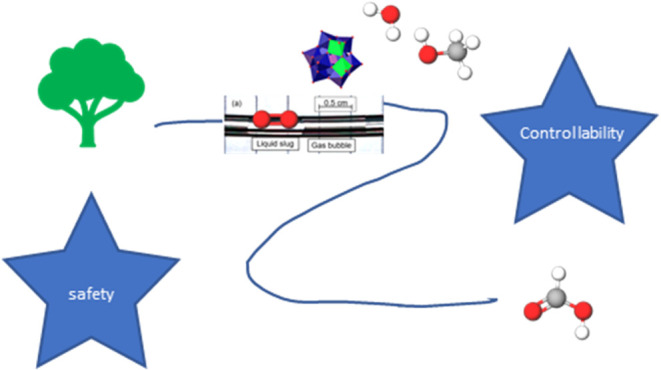

## Introduction

As the demand for energy continues to rise and the availability of fossil fuels diminishes, coupled with their adverse impact on the climate, the development of alternative carbon sources and fuels becomes imperative.^1^ Biomass is a renewable, carbon-neutral resource to produce energy and platform chemicals and, therefore, a promising alternative feedstock.^2,3^ It has the potential to contribute significantly toward sustainable energy development and the reduction of greenhouse gas emissions.^4^ Given that biomass encompasses a broad range of materials—including plant matter, agricultural crops, animal wastes, and the organic content of municipal and domestic waste—it is essential to make clear distinctions between these different types.^5^ First-generation biomass comprises crops (e.g., carbohydrates) and is mainly used for nutrition purposes.^6^ Here, glucose is a very common example of various biomass valorization projects despite being a critical part of nutrition. Therefore, it is better to use at least second-generation biomass, which contains agricultural and other lignocellulosic biomass.^6^ In particular, C5 sugars (e.g., xylose) derived from hemicellulose are interesting substrates, as they are produced in large quantities as residues, but can hardly be utilized by e.g., competitive biotechnological processes.^7^

In general, the valorization of biomass can proceed via thermochemical processes (e.g., pyrolysis, gasification),^8,9^ sono- and mechanochemical activation,^10^ biochemical conversion (e.g., fermentation)^11^ or chemo-catalytic processes (e.g., hydrolysis, oxidation or hydrogenation).^12,13^ The resulting products can be used directly or can be converted to value-added chemicals.^14–16^ One example is catalytic biomass oxidation to formic acid (FA).^17^ Today, FA is still produced from fossil resources and is used in various industries, including textiles and pharmaceuticals. The application of FA as a chemical hydrogen carrier of the future and the production of hydrogen from FA are important research topics.^18–21^ Biomass oxidation in the so-called OxFA process enables the conversion of a diverse range of biomass into FA and CO_2_ under mild reaction conditions.^22^ For instance, it is possible to utilize the biomass hydrolysate Renmatix derived from the Plantrose process for the production of FA.^23^

In the OxFA process, the oxidation reaction is catalyzed by vanadium-substituted phosphomolybdates derived from the class of polyoxometalates (POMs), utilizing molecular oxygen as an oxidant and water as an environmentally benign solvent.^24^ The chemical pathways of biomass oxidation have already been extensively studied.^25–27^ The catalyst oxidizes the biomass substrate and is simultaneously reduced from V^5+^ to V^4+^.^28^ The subsequent reoxidation and regeneration of the catalyst (from V^4+^ to V^5+^) is achieved through the use of molecular oxygen. The latter can be investigated by using ultraviolet–visible (UV–vis) spectroscopy.^23^ It is known that the rate-limiting step is the catalyst reoxidation. In an aqueous solution, typically high substrate conversion with FA yields of 50–60% and CO_2_ yields of 30% are reached.^30^

For the state-of-the-art OxFA process in aqueous media, mostly stirred-tank reactors (STR) have been used due to their efficient gas entrainment with optimized stirrers, and the option to use biomass substrates in diverse phases and shapes (such as solids, liquids, or suspensions).^22,23,26^ Here, batch, semibatch and continuous operation modes have been investigated.^24–30^ In 2019, Ponce et al. introduced a liquid core waveguide membrane microreactor allowing in situ spectroscopy with a high gas-to-liquid mass transfer through the used Teflon AF tube, yielding valuable knowledge about the redox kinetics of the used POM catalyst.^23,25^ Drawbacks of STR reactors are the mass transfer limitation caused by low interfacial surface area as well as long reaction times and insufficient reoxidation of the POM catalyst.^21,30,35,36^

In the modified OxFA process, organic additives, such as methanol, ethanol, *n*- and *iso*-propanol, and DMSO, have been shown to significantly increase FA yield and selectivity and corresponding esters, while inhibiting carbon dioxide formation.^13,37–39^ This is attributed to the formation of hydrogen bonds, interacting with the catalyst in a positive way or as a radical scavenger. Yet, methanol shows to be the most promising additive concerning practicability, stability, and productivity.^37,39^

The introduction of volatile organic solvents, such as methanol, to oxidizing agents like oxygen or air requires special attention to safety aspects, especially excluding the potential explosion risk when organic solvents come in contact with an oxidative atmosphere. Therefore, a detailed hazard operation study revealing the critical reaction parameters was carried out. Based on this, all safety-relevant experiments have to be performed below the lower explosion limit (i.e., 5 vol % gaseous methanol in oxygen) using water as the continuous liquid phase in high access. In comparison to STRs, microreactor setups demonstrate superior intrinsic safety properties. Characterized by the absence of moveable parts that could act as an ignition source and internal channel diameters ranging from a few to hundreds of micrometers, microreactors facilitate optimal mass and heat transfer, preventing the formation of dead zones and minimizing the accumulation of an explosive gas phase. Additionally, the minimization of local hot spots, which could otherwise serve as ignition sources for thermal explosions, is achieved. Due to the optimized gas–liquid mass transfer in microreactors, only small amounts of organic additives up to 10 vol % in water are required.^35,37^ Water can act as an additional heat scavenger and transfer agent. Consequently, it will be possible to operate far below the lower explosion limit at all investigated temperatures beneath 120 °C. This has the further advantage that undesired thermally induced side reactions of the biomass feedstock can be elegantly suppressed.

For homogeneous gas–liquid oxidation reactions, a Taylor-flow reactor can be a suitable microreactor setup.^29,30^ The so-called Taylor flow is characterized by alternating gas (Taylor) bubbles and liquid slugs flowing through microchannels.^31–34^ This flow pattern creates a large interfacial area and thereby increases the mass transfer of the gaseous compounds into the liquid phase.^39^ The increased intrinsic stirring of the liquid within the bubbles leads to boundary renewal, further contributing to the increased mass transfer.^35^ Characteristic numbers are the mass transfer coefficient (*k*_L_ *a*) or the Hatta number (*Ha*), providing insights into the interplay between mass transfer and reaction kinetics, thereby enabling the optimization of the reactor design and operating conditions. It is of particular interest to determine the effectiveness of gas distribution into solution, as this exerts a direct influence on the catalyst performance.

Until now, microchannel reactors were designed for high flow rates and short residence times, which are not well suited for biomass oxidation reactions like the OxFA process, as these typically require residence times between 10 min and 24 h for reaching high biomass conversion at low temperatures.^31,36^ A new conceptualization compared to previously presented Taylor-flow concepts is the use of Coriolis Mass Flow meters supplying liquids from pressurized tanks, in contrast to HPLC pumps.^41,46,47^ Another aspect is the mixing and supply of the reaction solution to the reactor. Separation of the catalyst and substrate solution is beneficial and allows certain flexibility in setting up the reaction. It also ensures that no reaction takes place in the storage container and avoids the formation of concentration gradients. The efficient preparation of reaction solutions is done by mixing substrate and catalyst solution in a tube-in-tube T-junction, as shown in the literature.^31–34^ The Taylor flow is generated by mixing gas supplied by Mass Flow Controllers with the reaction solution in another T-junction.

The aim of this study is to investigate the suitability of a microreactor operated in Taylor-flow mode for the oxidation of the attractive biomass substrate xylose in the methanol-modified OxFA process under mild reaction conditions with temperatures below 120 °C to avoid any formic acid decomposition or thermally induced side reactions. Herein, it will be revealed whether Taylor flow leads to a better mass transport of oxygen, and thus enabling an easier reoxidation of the POM catalyst to achieve higher activities and FA yields. Moreover, a direct comparison to a classical STR will be made for both the model substrate and a commercial C5-sugar source.

## Results and Discussion

### Investigation of Hydrodynamics and Oxygen Solubility in the Taylor-Flow Microreactor

A possible advantage of Taylor-flow microreactors in the oxidation of C5-containing biomass is the improved oxygen solubility, which could increase the rate-limiting step of the POM catalyst reoxidation. Therefore, the characteristics of the Taylor-flow mode in the newly built reactor setup (Figure S1) and the associated oxygen solubility are investigated first. For this purpose, PFA tubes with different lengths and an optical oxygen sensor were used to determine the *k*_L_ *a* values for oxygen in the system and to observe the formation of the Taylor flow. The PFA tubes were installed directly after the T-junction. Figure 1a shows the successful formation of a dewetted slug flow for an equal gas and liquid volume flow (ε_G_ = 0.5), meaning that no liquid film was observable between the gas bubble and PFA tubing. Whereas wetted slug flow (liquid film around gas bubble) would be beneficial for improved mass transfer, dewetted slug flow provides unit cells of gas bubbles and liquid slugs, and therefore, no demixing is to be expected.

**Figure 1:**
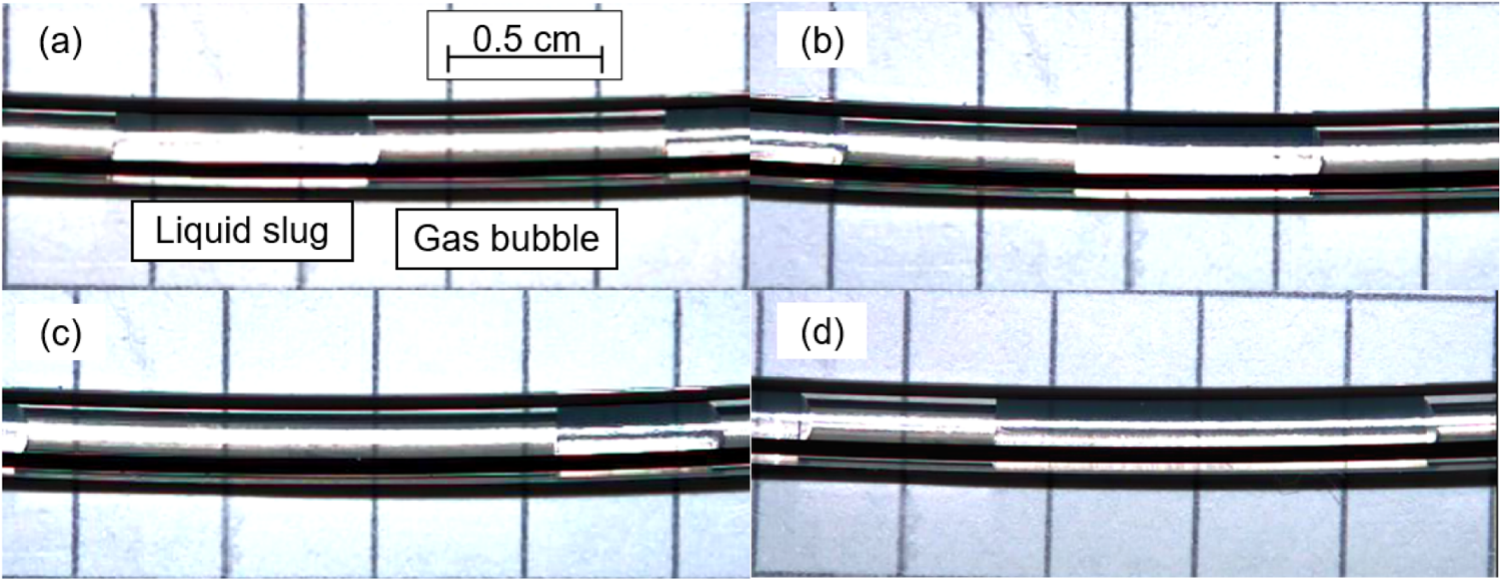
Variation of gas hold-up showing dewetted slug flow formation. (a) Gas volume flow = 1 mL min^–1^, liquid volume flow = 1 mL min^–1^, ε_G_ = 0.5, (b) gas volume flow = 4 mL min^–1^, liquid volume flow = 4 mL min^–1^, ε_G_ = 0.5, (c) gas volume flow = 3 mL min^–1^, liquid volume flow = 1 mL min^–1^, ε_G_ = 0.75, and (d) gas volume flow = 0.67 mL min^–1^, liquid volume flow = 2 mL min^–1^, ε_G_ = 0.25.

Visibly, higher velocity does not change slug length but only bubble/slug shape (Figure 1b). Lower velocity has a more pronounced spherical contact area, leading to a higher phase change surface. At a higher velocity the back pressure regulator had a higher influence on the formation of slug flow at the gas/liquid mixing T-junction due to the pressure fluctuations induced by liquid slugs leaving the system. Slug flow was also achieved by variation of gas hold-up, as visible in Figure 1c,d. Table S1 shows detailed measurements for the liquid slug and bubble lengths for various gas hold-ups.

In the following, the volumetric mass transfer coefficient (*k*_L_ *a*) and its implications for possible mass transfer limitations are investigated for the solvent water. Therefore, a variation of gas hold-up (εG, Figure 2a) and two-phase velocities (*u*_TP_, Figure 2b) versus measured volumetric mass transfer coefficient is shown together with calculated *k*_L_ *a* values using empirical models from the literature for the presented system. In Tables S1 and S2, the necessary parameters and assumptions for the calculations are summarized.

**Figure 2:**
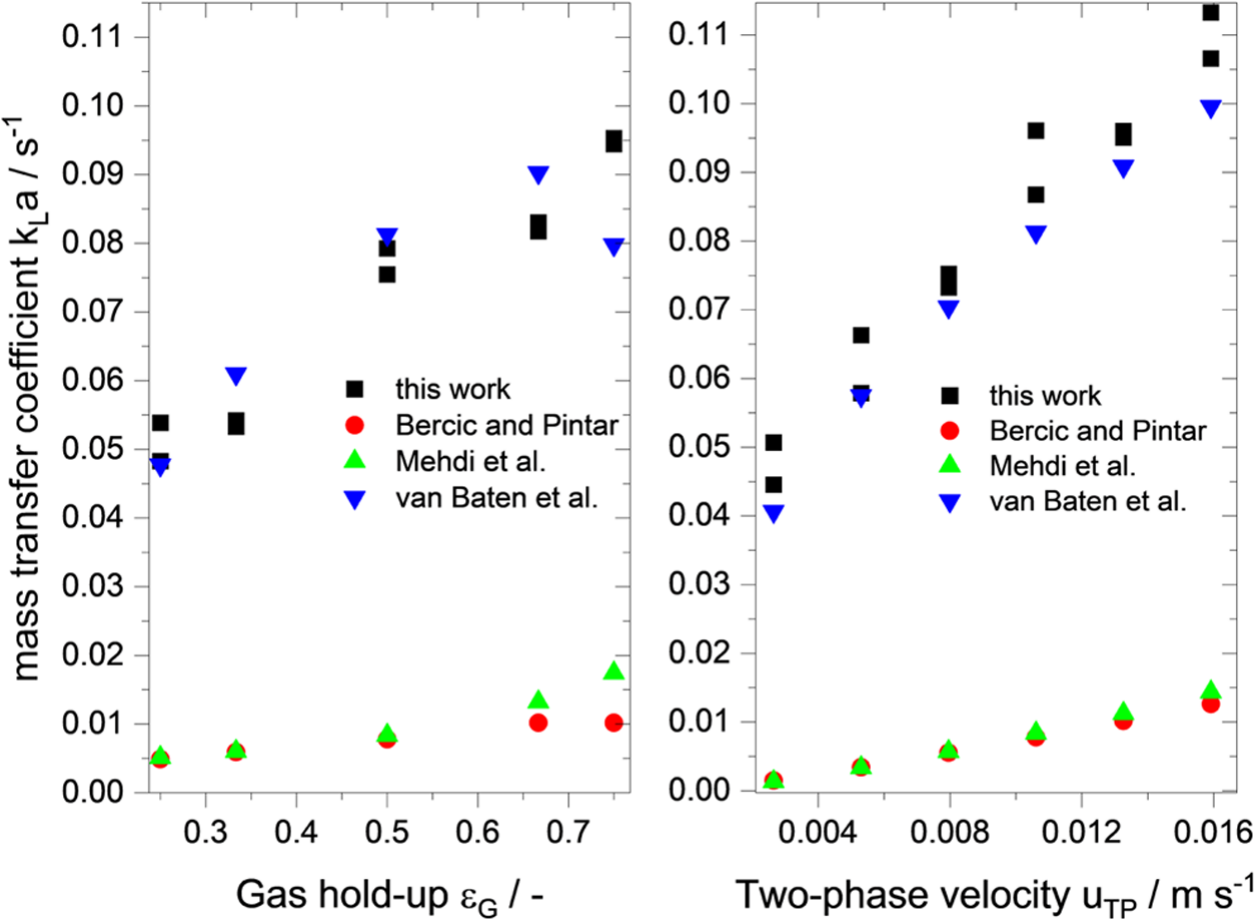
Volumetric mass transfer coefficient *k*_L_ *a* versus gas hold-up ε_G_ (left panel) and two-phase velocity *u*_TP_ (right panel). Measurement conditions: solvent: water, gas: nitrogen and synthetic air, *p*_total_ = 9 bar, *p*_O2_ = 0.525 bar, τ = 10 s, *l*_SS_ = 6 cm, *l*_PFA_ = 6 cm, *T* = 21 °C, *V*_total_ = 2 mL min^–1^ (left panel), and ε_G_ = 0.5 (right panel). Values calculated according to the literature.^37–42^

Gas hold-up is the dominant factor determining the bubble and liquid slug lengths in the respective setup and shows a strong influence on measured *k*_L_ *a* values. High *k*_L_ *a* values are achieved for long bubbles and short liquid slugs due to increased circulation and mixing inside the liquid slug. This is also true for increasing the two-phase velocity values. Here, high *k*_L_ *a* values are achieved with the highest two-phase velocities measured, although a dewetted slug flow is present in the PFA tube. These values indicate that the cap mass transfer mechanism exhibits a high degree of strength, compensating for the absence of film transfer. The shown data are in line with the calculated values by the model from van Baten et al.^43^ The empirical models by Bercic and Pintar and Mehdi et al. show the same trend, despite a strong deviation. These models were constructed using much faster flow velocities (0.018–0.45 m s^–1^), explaining the strong underestimation of the experimental values.^37,40^ To determine the effect of the additive methanol, solutions containing 5 vol %, respectively, 10 vol % methanol in water were added and additional experiments with different two-phase velocities were conducted to achieve corresponding *k*_L_ *a* values (see Figure S6).

The addition of 5 vol % methanol leads to a slightly higher *k*_L_ *a* value of 0.042 s^–1^ at a *u*_TP_ of 0.0027 m s^–1^ compared to pure water with a *k*_L_ *a* value of 0.040 s^–1^. For both methanolic solutions, increasing *k*_L_ *a* values were observed for higher velocities. At the highest tested u_TP_ of 0.016 m s^–1^ the methanolic solutions reached a *k*_L_ *a* value of 0.10 s^–1^ compared to the aqueous *k*_L_ *a* value of 0.085 s^–1^. This is related to an increased oxygen solubility in methanolic aqueous solutions.^44^ To assess its efficiency, a comparison of the gas–liquid mass transfer was made with other reactor concepts. The presented system shows *k*_L_ *a* values ranging from 0.04 to 0.1 s^–1^, which are in the same range as bubble column reactors (0.1–1 s^–1^) and significantly higher than STRs (0.01–0.02 s^–1^).^45^ This is an interesting point for future reaction engineering optimization, as lower pressure would be needed to achieve the same amount of gas dissolved inside the liquid bubbles compared to STRs.

The correlation between mass transfer limitations and intrinsic reaction kinetics can also be evaluated by using the Hatta number (*Ha*). This dimensionless number is used for homogeneous catalytic reactions in biphasic systems and describes the ratio of the reaction time *t*_r_ to the characteristic time for mass transfer *t*_m_ into the liquid phase. Since the reaction only takes place in the liquid phase, the reactant oxygen must first transfer from the gas into the liquid phase. The reaction rate in relation to the mass transfer velocity defines whether the reaction mainly takes place near the interface or in the bulk of the liquid phase. The following assumptions were drawn to evaluate this scenario: (a) the reaction is irreversible, (b) the mass transfer resistance in the nonreactive gas phase is negligible, and (c) the substrate and the products have negligible concentrations in the gas phase. Due to the dewetted slug flow, only the bubble cap area *a*_cap_ was considered for calculations. Hatta numbers for different velocities in the microreactor system can be found in Table 1. The calculations were performed according to eq 4 shown in the Experimental Details section. Shown values far below the threshold of *Ha* = 0.3 indicate the absence of mass transfer influences in the microreactor system presented here for the investigated oxygen pressures.

**Table 1:** Hatta Number Calculated for the Experimental *k*_L_ *a* Values in the Setup with *l*_tube_ = 12 cm and *d*_c_ = 2 mm^a^

*u*_TP_/m s^–1^	(*k*_L_ *a*)_exp_/s^–1^	area of the bubble cap *a*_cap_/m^2^ m^–3^	(*Ha*)_cap_/-
0.00265	0.0394	250	0.06
0.00531	0.0599	250	0.04
0.00796	0.0716	250	0.03
0.1061	0.0744	250	0.03
0.1326	0.0843	250	0.03
0.01592	0.0882	250	0.03

a Reaction conditions: *T* = 100 °C, *k*_ox_ = 0.2965 min^–1^, *k*_red_ = 0.9763 min^–1^, *c*_HPA‑5,tot_ = 0.012 mol L^–1^, *D*_O2,L_ = 2 × 10^–9^ m^2^ s^–1^, ε_G_ = 0.5, *L*_UC_ = 0.016 m.^25^

### Comparing Xylose Oxidation in Pure Aqueous and Aqueous-Methanolic Solution

The results show that a Taylor flow was successfully formed in the microreactor setup. Following the successful hydrodynamic characterization using PFA tubing, the first results of xylose oxidation from the microreactor in the stainless-steel tubes are presented. To test the suitability of the system for xylose oxidation, benchmark xylose oxidation reactions were performed under standard reaction conditions (*T* = 100 °C and *p*_O2_ = 25 bar) in pure water (100% H_2_O) and with the additive methanol in small amounts of 10 vol % (90:10 vol % H_2_O/MeOH).^17,26,28^ As the reaction temperature has a high influence on oxidative sugar conversion, the heat transfer properties of the used heating bath into the tubular reactors were investigated. Within Taylor flow conditions after 10 s residence time, the oil bath temperature was reached in the outlet (see Figure S7). The residence time was varied by using different tube lengths (*V*_1_ = 15 mL, *V*_2_ = 30 mL, *V*_3_ = 60 mL, and *V*_4_ = 120 mL). This allowed the flow velocities of the gas and liquid phases to be kept uniform to not affect mass transfer any further. In order to evaluate the performance of the reactor, results for xylose conversion from STR reactions under similar reaction conditions were also obtained. Sampling times were in the region of residence times in the microreactor setup. Figure 3 shows the results for xylose oxidation in 100% H_2_O (Figure 3a) as well as 90:10 vol % H_2_O/MeOH (Figure 3b) for both reactor concepts (Taylor-Flow microreactor vs STR).

**Figure 3:**
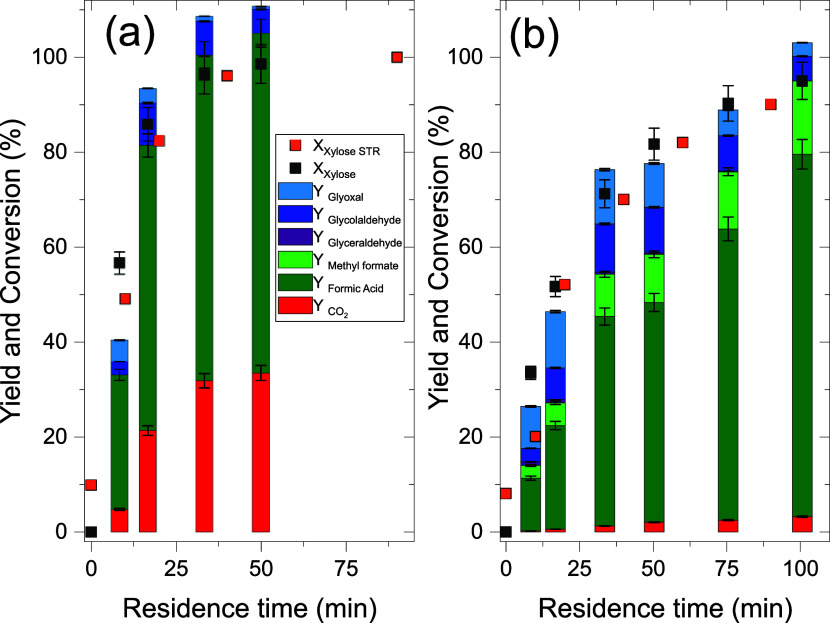
Comparison of STR and the Taylor-flow microreactor for aqueous xylose oxidation. Reaction conditions: *c*(Xylose) = 50 mmol/L, *c*(HPA-5) = 12 mmol/L, *p*_O2_ = 25 bar, *T* = 100 °C, solvent: (a) H_2_O , (b) 90:10 vol % H_2_O/MeOH ; for Taylor-flow plant: ε_g_ = 0.5, reaction times were set by reactor length choice and total volumetric flow rate, *n* = 3; for STR: *V* = 45 mL, and *v*_stirrer_ = 1000 rpm (with gas entrainment).

With increasing residence time, the conversion of xylose increases for both investigated reactor systems. After 30 min of reaction time, full conversion for xylose was obtained for pure water (Figure 3 (a)). Prolonging the residence time to 50 min does not show significant changes. The product distribution shows that approximately 70% FA and 30% CO_2_ are present at full conversion. Small amounts of glycolaldehyde are still present after 50 min. The yield of over 100% can be related to minor inaccuracies in HPLC yield determination, summing up to 10%. The intermediates glyoxal and glyceraldehyde are present in minor amounts, each accounting for less than 5%, whereas glycolaldehyde reaches a maximum yield of 17% after 20 min.

Xylose conversion with the methanol-containing solution is significantly slower compared to the reaction in pure water (Figure 3 (b)). For 90% xylose conversion, a residence time of 70 min was needed. Formation and conversion of intermediates are also changed in the methanolic system when compared to the experiments in pure water. Glycolaldehyde and glyoxal are the only intermediates showing significant yields at residence times of up to 100 min. Both are slowly converted, showing yields of 5% after 100 min of reaction. Carbon dioxide formation is suppressed to a yield below 5%. As demonstrated in Figure S8, the methanolic system could be successfully reproduced three times. The xylose conversions are very similar to the obtained STR results for both systems. The differences in the 0-min samples can be attributed to the different heating behavior of the STR . A small amount of xylose was already converted during heating from room temperature to the desired reaction temperature of 100°C. Nevertheless, both reactor setups show similar results in both reaction modes with comparable yield and conversion, indicating that the batch oxidation reaction could be effectively transferred into a continuous microreactor system. To ascertain whether the microreactor setup exerted a detrimental effect on the structure of the catalyst, ^51^V-NMR spectra were recorded prior to and following the reaction. However, the studies revealed no alterations in the spectra of the catalyst either during the experiment (corresponding spectra can be found in Figure S9). As demonstrated in Figure S8, the dark orange to red color obtained for all liquid samples during the oxidation experiments indicates that mass transfer limitations do not play a major role here.

### Pressure Variation in a Taylor-Flow Microreactor in an Aqueous-Methanolic Solution

From an energetic perspective, operating at lower partial oxygen pressures would be advantageous. This is due to a decrease in construction costs owing to a lower total pressure of the oxidant. However, in the event of uncertainty, this approach would result in a significant reduction in oxygen mass transfer, which could adversely impact the catalyst reoxidation and consequently the overall reaction rate. Therefore, it was investigated whether a partial O_2_ pressure variation at fixed reaction times influences xylose conversion (Figure 4).

**Figure 4:**
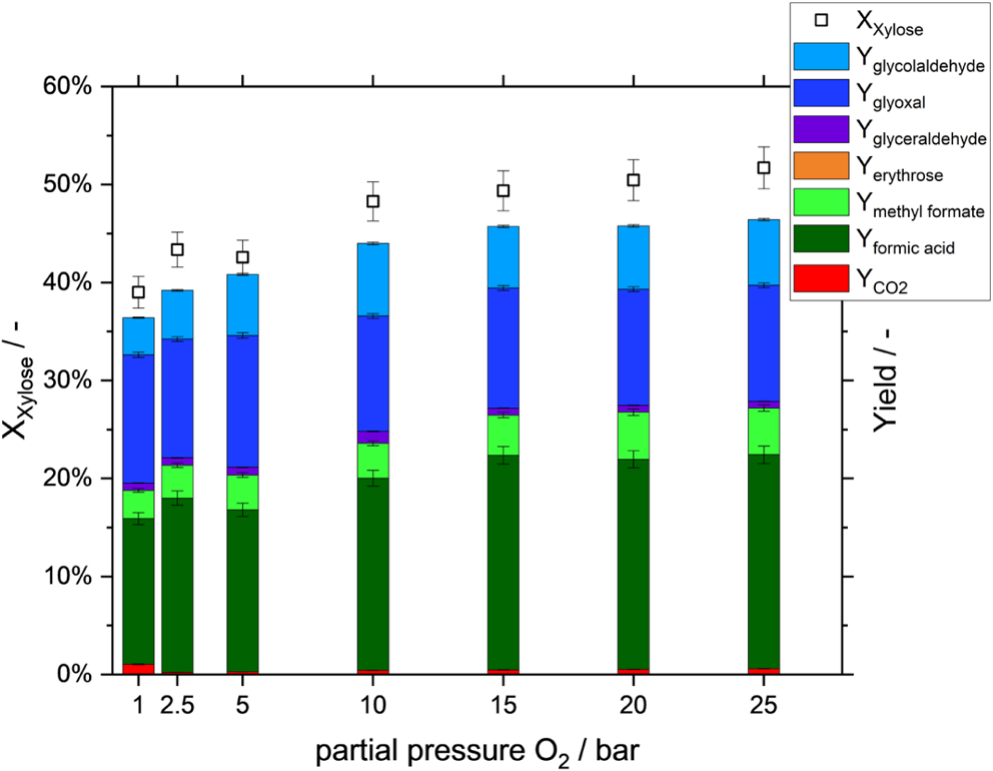
Pressure variation in an aqueous-methanolic solution for fixed reaction time inthe Taylor-flow microreactor. Conversion and yields versus total/partial oxygen pressure. Reaction conditions: *c*(Xylose) = 50 mmol/L, *c*(HPA-5) = 12 mmol/L, *T* = 100 °C, *p*_O2_ = variable/as shown, solvent composition: 90:10 vol % H_2_O/MeOH; ε_g_ = 0.58, τ = 16.8 min.

By comparing different partial pressures at a fixed residence time of 16.8 min, the impact of oxygen mass transfer can be deduced. Xylose conversion is increased from 40 to 47% as oxygen pressure increases from 1 to 10 bar, while it remains constant at higher pressures. This suggests that 10 bar *p*_O2_ is sufficient to facilitate rapid catalyst reoxidation without compromising xylose conversion. At lower pressures, the reduced oxygen concentration in the liquid phase is likely to become a limiting factor. However, the potential benefits of reduced presaturation effort for oxygen could counterbalance the slight decline in conversion efficiency. As documented in the literature, the OxFA process can be performed using compressed air rather than pure oxygen, while maintaining an identical oxygen partial pressure (assuming 20% O_2_ in air).^24^ This approach is viable only if the total pressure remains at a low level, even with air, thereby circumventing the need for costly air separation. The yields of the products and intermediates appear to be unaffected by the oxygen pressure. This phenomenon can be attributed to the observation that the reoxidation of the catalyst, being the slowest reaction step in the performed reaction at pressures below 10 bar *p*_O2_, is not significantly impacted by alterations in oxygen pressure (see Figure 5).^21^ This can be explained by the fact that within the study presented here, the substrate oxidation is the slowest reaction step at lower oxygen partial pressure.

**Figure 5:**
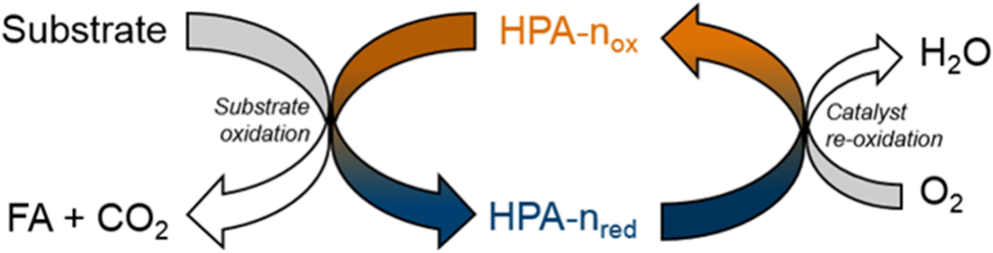
General catalytic scheme of the OxFA process divided into substrate oxidation (left) and catalyst reoxidation (right).^24^

Figure 6 displays the xylose conversion at different partial pressures of oxygen in the first 40 min reaction time (left) compared to extinction values measured at 750 nm for semiquantitative determination of the V^4+^ content. Complete yield and conversion values can be found in Figures S11–S15.

**Figure 6:**
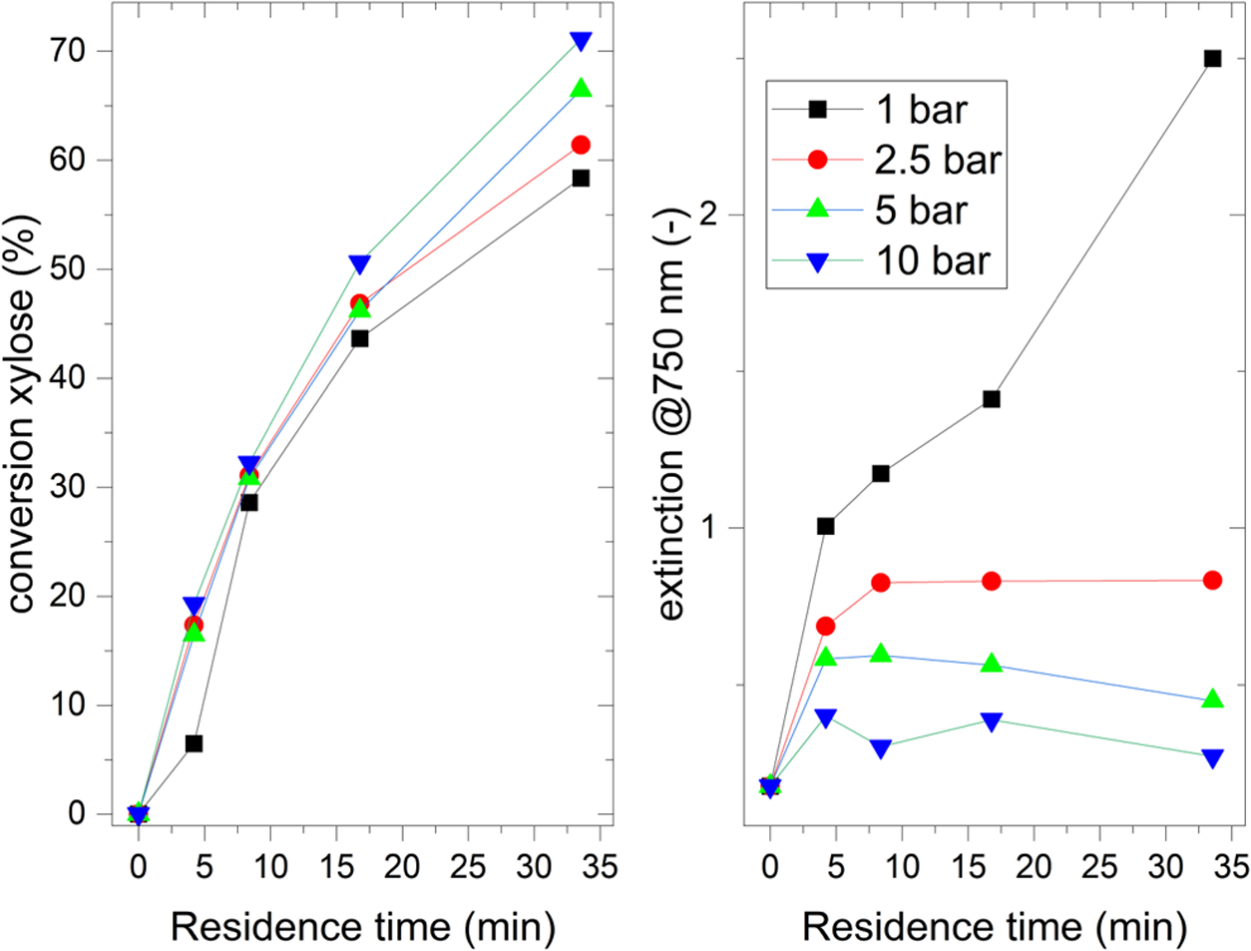
Xylose conversion (left panel) and corresponding extinction values at 750 nm for V^4+^ content determination (right panel) achieved by partial oxygen pressure variation in an aqueous-methanolic system over reaction time inthe Taylor-flow microreactor. Reaction conditions: *c*(Xylose) = 50 mmol/L, *c*(HPA-5) = 12 mmol/L, *T* = 100 °C, solvent composition: 90:10 vol % H_2_O/MeOH; ε_g_ = 0.58, reaction times were set by reactor length choice.

An increase in xylose conversion was observed for all tested partial oxygen pressures with longer residence times (left). Until 10 min of residence time, the xylose conversion did not vary. However, with longer residence times, the reaction performed with 1 bar of partial oxygen pressure showed significantly lower conversion values compared to the higher partial oxygen pressures. At the longest residence time of 36 min, a variation of 15% xylose conversion was obtained, with the highest value being paired with the highest oxygen partial pressure and vice versa. Again, the yields of the different products appear to be unaffected by the oxygen pressure. Therefore, it can be assumed that the low partial oxygen pressure indeed has a negative influence on the xylose conversion.

In order to provide further validation, the reaction solutions were analyzed by UV–vis spectroscopy (Figure S10). The extinction values at 750 nm were found to correlate, in a semiquantitative manner, with the content of reduced V^4+^ species in the reaction solution (see Figure 6 (right)).^23^ At the start of the reaction (*t* = 0), the catalyst was partly reduced (extinction of 0.2). After a residence time of 4.6 min, an increase in these values was observed for all partial O_2_ pressures tested. The higher pressures (5 and 10 bar) show a maximum extinction of 0.6 (5 bar) and 0.4 (10 bar) with a slight decrease in values as the residence time increases. At 2.5 bar, the influence is less pronounced, with a slight increase in absorbance after the first measurement up to an extinction of 0.8 and then remaining constant. At 1 bar O_2_ pressure, the vanadium is predominantly in the reduced V^4+^ state and shows an increase in extinction value up to 2.5 with prolonging reaction time, indicating a challenge in catalyst reoxidation, as previously discussed in other microreactor studies.^29^ Interestingly, this has a high impact on xylose conversion, lowering the values by 10% compared to higher partial oxygen pressures at the same residence time of 35 min. Due to the low partial oxygen pressure, the content of dissolved oxygen in the reaction solution is reduced to such an extent that the rate of catalyst reoxidation is also reduced and thus dominates the entire catalytic cycle (Figure 5).

To further investigate this phenomenon, an additional gas hold-up variation experiment was performed at low O_2_ pressures (see Figure 7, full results, including product distribution, can be found in Figure S16). It is speculated that increasing gas hold-up would increase mass transport, as indicated by the findings of hydrodynamic investigations conducted above.

**Figure 7:**
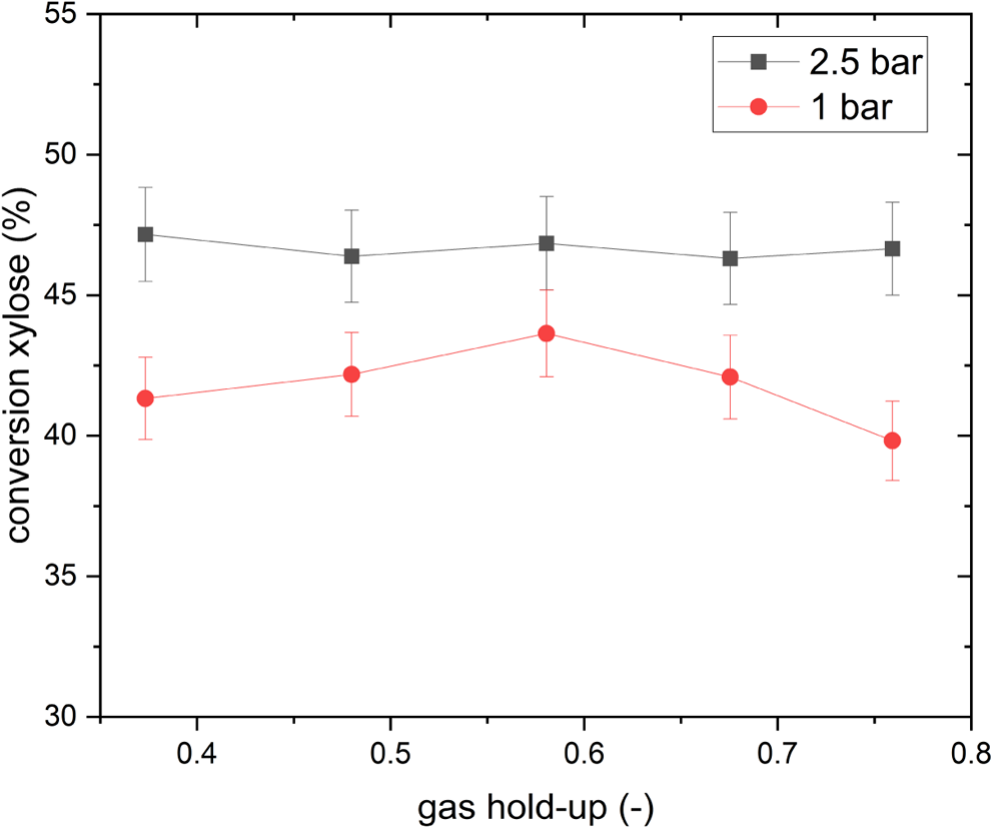
Variation of gas hold-up at 2.5 and 1 bar partial O_2_ pressure and a residence time of 16.8 min. Reaction conditions: *c*(Xylose) = 50 mmol/L, c­(HPA-5) = 12 mmol/L, *T* = 100 °C, solvent composition: 90:10 vol % H_2_O/MeOH; τ = 16.8 min.

However, with an increased gas hold-up, no difference in xylose conversion for both investigated partial oxygen pressures was observed. Conversion values of xylose for the 1 bar experiment are lower compared to those for the 2.5 bar experiment, as observed beforehand. This is also true for the product and intermediate yield distributions. Furthermore, according to hydrodynamic investigations performed above, mass transport is strongly influenced by the two-phase velocity.^46^ Therefore, a variation of two-phase velocities at 2.5 bar p_O2_ was performed for a shorter residence time of 8.4 min (results see Figure S17). Hardly any changes in xylose conversion with increased phase velocities could be observed. It can thus be concluded that an increase in gas hold-up and two-phase velocity does not result in an increase in mass transfer/oxygen availability within the liquid bubble for a faster catalyst reoxidation. Consequently, this does not exert any further influence on xylose conversion. Contrary to the initial suppositions, it is postulated that, owing to dewetted slug flow within the system, the exchange surface is predominantly the rounding at the anterior and posterior of the slug, as opposed to the liquid film on the tube wall.

This is even though the employed stainless-steel tubing exhibits a reduced contact angle between the liquid and the wall in comparison to the examined PFA tubing, thereby theoretically enhancing the wetting behavior.^47,48^

### Experiments with Renmatix as Exemplary Complex Biomass in the Taylor-Flow Microreactor in an Aqueous-Methanolic Solution

Renmatix is an aqueous solution of xylose generated from partially hydrolyzed wood in the Plantrose process. It contains 20–30% crude xylose, <10% other carbohydrates and lignin, <1% acetic acid, formic acid, furfural, and 5-hydroxymethylfurfural, and it is completely soluble in water/methanol mixtures containing no macroscopic particles.^41^

Figure 8 shows the results when using Renmatix as a real biomass substrate. The substrate concentration is normalized to the same xylose concentration values as in the previously shown reactions (*c*_xylose_ = 50 mmol/L) with reaction conditions as before.

**Figure 8:**
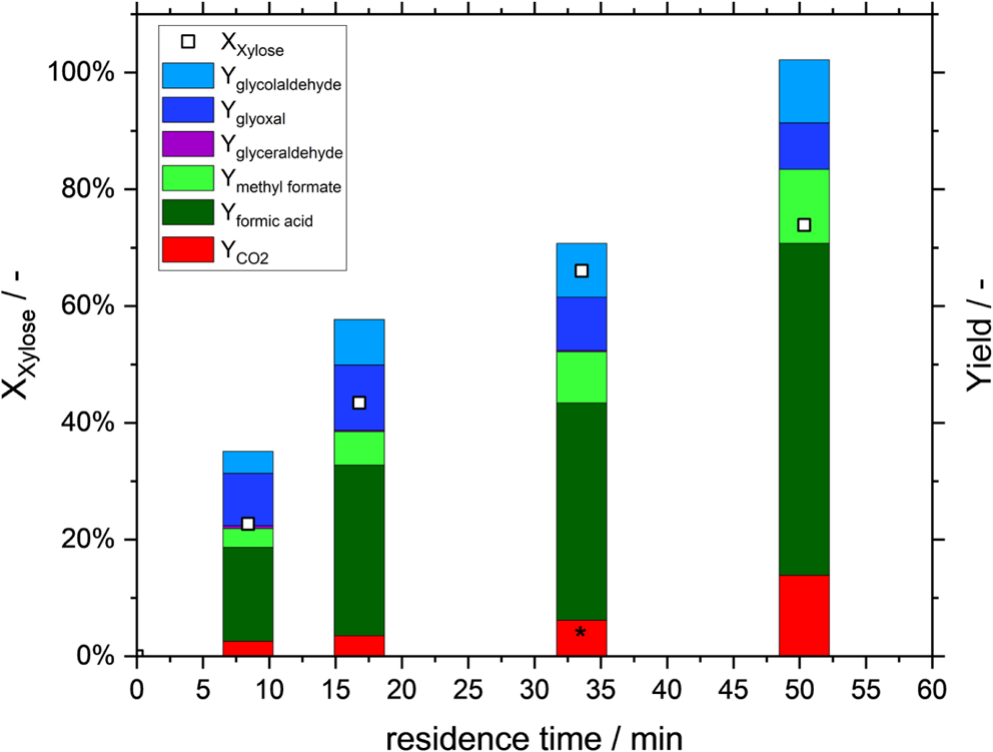
Variation of residence time and Renmatix substrate in the Taylor-flow microreactor. Reaction conditions: *c*(Xylose) = 50 mmol/L, *c*(HPA-5) = 12 mmol/L, *T* = 100 °C, solvent composition: 90:10 vol % H_2_O/MeOH; *p*_O2_ = 10 bar. ε_G_ = 0.58; *CO_2_-Yield assumed based on ratio of CO_2_/FA in other data points.

Once more, an increase in xylose conversion is observable in instances of elevated residence times. Following a period of 50 min, a xylose conversion of 80% is attained, a result that is analogous to those obtained with the model substrate xylose (cf. Figure 3). Figure S18 shows a well-working microreactor setup with real biomass substrate conversion for a residence time variation by using a partial oxygen pressure of 2.5 bar. Also, no formation of solid particles within the systems could be observed, which would decrease the efficiency of the system. The presented yields are associated with the xylose content, thereby elucidating the overestimated carbon balance. The comparatively elevated CO_2_ yields, despite the utilization of methanol as an additive, can be attributed to the employment of a genuine biomass substrate. Nevertheless, the yields of the intermediates glycolaldehyde and glyoxal as well as the formation of formic acid and methyl formate, show that the conversion of real biomass hydrolysate is possible within the Taylor-Flow microreactor setup presented here under very mild reaction conditions.

## Conclusions

In the modified OxFA process, various biogenic substrates can be selectively oxidized to the sustainable hydrogen carrier formic acid using the H_8_PV_5_Mo_7_O_40_ (HPA-5) POM catalyst in an aqueous-methanolic solution with molecular oxygen or compressed air as an oxidant. A current challenge of the mainly used stirred-tank reactors is the mass transfer limitation with respect to the effective dissolution of oxygen in the reaction media. In order to improve the gas–liquid mass transfer, alternative reactor concepts are needed. The successful application of a Taylor-flow microreactor for the catalytic oxidation of xylose and the commercial C5-sugar Renmatix, both for the OxFA process in pure water and the modified OxFA process in an aqueous-methanolic solution highlights its potential as a robust and efficient method for continuous biomass oxidation. The process demonstrates high FA yields up to 90% within 100 min for xylose and 70% for Renmatix under mild reaction conditions of 100 °C and 25 bar oxygen pressure, outperforming alternative methods reported in Taylor-flow studies involving glucose. This success can be attributed to the system’s capacity for efficient oxygen mass transfer across diverse reaction and flow conditions, emphasizing its viability for scalable and energy-efficient biomass processing.

## Experimental Details

### Materials and Catalyst Synthesis

All chemicals were obtained commercially and used as received without further purification. d­(+)-xylose (99%) and methanol (99.8%) were supplied by Merck KGaA and VWR BDH Chemicals. The biomass hydrolysate Renmatix was supplied by OxFA GmbH. The vanadium-substituted polyoxometalate catalyst HPA-5 (H_8_PV_5_Mo_7_O_40_) was supplied by OxFA GmbH as a 6.2 wt % aqueous solution for the Taylor-flow microreactor experiments or was synthesized as previously described for experiments carried out in the stirred-tank reactor.^49–51^ Nitrogen, synthetic air, helium, and oxygen (5.0) were supplied by Air Liquide.

### Catalyst Characterization

The characterization of the HPA-5 catalyst was carried out using a Fa. Spectro Arcos ICP-OES device resulting in a Mo/P/V ratio of 7:1.3:4.8. The desired Keggin-structure type was verified by FT-IR spectroscopy using an IRSpirit-X equipped with an ATR unit from Shimadzu (Figure S3). The UV–vis measurements analyzing the reaction solutions from the microreactor were performed using a SPECORD UV–vis Spektralphotometer by Analytik Jena. The catalyst within the reaction solutions was analyzed further by ^51^V- and ^31^P-NMR spectroscopy using a Bruker Avance III HD 600 MHz spectrometer of the central analytics department of the University of Hamburg.

### Characterization of the Used Taylor-Flow Microreactor

The flow sheet of the self-constructed microchannel reactor setup can be found in Figure S1. The plant consists of four main sections, which are defined as (A) gas supply, (B) liquid supply, (C) tubular reactors immersed in a heated oil bath, and (D) the sample loop together with a downstream waste container. Catalyst and substrate solutions are dosed by Bronkhorst mini Cori-Flow Coriolis mass flow meters and mixed in a tube-in-tube T-junction. The gas flow is controlled by Bronkhorst mass flow controllers using an El-Flow Select type for oxygen and nitrogen and an El-Flow Prestige type for helium. The gas phase is introduced by line in line via a T-junction into the premixed catalyst–substrate solution, setting up the Taylor flow before entering the reactor. The setup is equipped with four tubular reactors of different lengths and equal volumes of 15, 30, 60, and 120 mL, respectively. The tubular reactors (1/8″) are coiled and fully submerged in a heating bath, which is filled with silicone oil and regulated by a Huber Unistat Tango circulation thermostat. A reactor tube of a certain length can be chosen by adjusting the respective ball-valves. Close to the outlet, the tubes are combined and cooled to 7 °C, using a tube-in-tube cooler to allow sampling of the highly volatile products. Afterward, the reaction solution either flows through the sample loop or reaches the Equilibar differential back pressure regulator (Alicat, PCS-DV-A-70PG-AG). All parts are constructed from 316 stainless steel and have been cleaned with iso-propanol to ensure that no oxidizable content remains prior to the introduction of high-pressure oxygen.

### Analysis of Taylor-Flow Formation and Stability

For optical verification of Taylor-flow formation under different conditions, a perfluoroalkoxy (PFA) tube with an inner diameter of 2 mm was installed at three different locations in the microreactor setup (Figure S1). These were located immediately downstream of the gas–liquid mixing T-junction, following the valves that selected the tubular reactor in use and directly upstream of the back pressure regulator. This was to ensure the prevention of gas–liquid phase separation throughout the system. A high-speed camera equipped with a high-speed microscope (Keyence VW-600C and Keyence VW-9000D) was used to record slow-motion videos of the Taylor flow at various settings (pictures in Figure 1).

### Analysis of Oxygen Mass Transfer

The rate of oxygen mass transfer into the liquid phase was analyzed using an optical oxygen mini-sensor from PyroScience combined with a FireSting fiber-optic oxygen meter. The measurements were conducted after two different lengths (7 and 12 cm) behind the T-junction for gas/liquid mixing. To achieve this, the sensor tip was positioned within the inlet PFA tube at a T-junction, ensuring that Taylor-flow disturbances occurred only downstream of the measurement point. This setup enabled the measurement of O_2_ concentration within both gas and liquid slugs. Immediately downstream, a temperature sensor was integrated into another T-junction. For quantification, the sensor was calibrated by using dry air (0% humidity) as the reference standard in conjunction with the recorded temperature. The calibration was validated by measuring air-saturated solutions of pure water, as well as 90:10 and 95:5 vol % water–methanol mixtures, and comparing the obtained values with literature data. An exemplary oxygen partial pressure graph within the Taylor-flow regime is shown in Figure S4.

The volumetric mass transfer coefficient *k*_L_ *a* was calculated by eq 1, considering the liquid equilibrium concentration c*_O2,L_, inlet and outlet concentration *c*_O2,L_^in/out^ and residence time τ.

1 $$k_{L}a=\frac{1}{\tau }\ln\left(\frac{c_{O2,L}^{*}-c_{O2,L}^{\mathrm{in}}}{c_{O2,L}^{*}-c_{O2,L}^{\mathrm{out}}}\right)$$

Oxygen concentration in liquid *c*_O2,L_^in^ was measured in the liquid inlet without any gas slugs at the beginning of each experiment. The outlet concentration *c*_O2,L_^out^ was measured at the liquid outlet under Taylor-flow conditions and averaged from at least 15 representative liquid slugs and their minimum liquid slug concentrations.

For calculation of the liquid equilibrium concentration c*_O2,L_ (see eq 2), the uncorrected oxygen gas concentration c*_O2,G,uncorr_ was measured by flushing the tubes with a gas mixture of nitrogen and synthetic air (*p* = 9 bar, *p*_O2_ = 0.502 bar). The reference liquid equilibrium concentration c*_O2,L,ref_ was measured by bubbling the gas mixture through the respective liquid until equilibrium was reached. The setup for determination of equilibrium concentrations can be seen in Figure S5. The reference gas concentration c*_O2,G,ref_ was measured under equal conditions by exhausting the gas mixture into an empty sample vial.

2 $$c_{O2,L}^{*}=c_{O2,G,\mathrm{uncorr}}^{*}\frac{c_{O2,L,\mathrm{ref}}^{*}}{c_{O2,G,\mathrm{ref}}^{*}}$$

The residence time τ was obtained from the total volumetric flow rate, as well as lengths and diameters of used stainless-steel tubing (*d*_SS_ = 1.75 mm) and PFA tubing (*d*_PFA_ = 2.00 mm), according to eq 3.

3 $$\tau =\frac{\frac{1}{4}\pi \left(l_{\mathrm{PFA}}d_{\mathrm{PFA}}^{2}+l_{\mathrm{SS}}d_{\mathrm{SS}}^{2}\right)}{\mathrm{V̇}}$$

The Hatta number was calculated by using eq 4.

4 $$\mathrm{Ha}=\sqrt{\frac{t_{m}}{t_{r}}}=\delta_{L}\sqrt{\frac{k_{\mathrm{ox}}{\left(c_{\mathrm{cat},\mathrm{red}}\right)}^{\alpha }{\left(c_{o_{2},L}^{*}\right)}^{\beta -1}}{D_{o_{2},L}}=\frac{\sqrt{k_{\mathrm{ox}}\cdot c_{\mathrm{HPA}-5,\mathrm{tot}}\frac{k_{\mathrm{red}}}{k_{\mathrm{red}}+k_{\mathrm{ox}}}Do_{2,L}}}{\frac{{\left(k_{L^{a}}\right)}_{\exp}}{a_{\mathrm{cap}}}}}$$

The calculation can be simplified by assuming a first-order reaction in oxygen concentration (β ≈ 1).^30^ In addition, Ponce et al. found the reaction order of HPA-5 during the reoxidation of the reduced catalyst to be 0.98 ≈ 1 = α.^25^ Due to the nearly constant concentration of reduced HPA species, a pseudo-first-order reaction can be assumed. At equilibrium of the reduction and reoxidation reactions, the respective reaction rates (*r*_red_ and *r*_ox_) are equal. Therefore, the concentration of reduced catalyst species (*c*_HPA,red_) is nearly constant and can be expressed as a function of the total catalyst concentration (*c*_HPA–5,tot_) and the respective reaction rate constants (*k*_red_ and *k*_ox_). The diffusion coefficient *D*_O2,L_ was taken from the literature.^52^ Due to the dewetted slug flow, only the area of the bubble caps, *a*_cap_, is considered. For the experimental *k*_L_ *a*-value, the average of two measurements taken in the 12 cm long setup in water was used.

### Catalytic Experiments in the Taylor-Flow Microreactor

For a typical experiment in the Taylor-flow microreactor, a substrate solution containing xylose (*c*_Xylose_ = 50 mmol/L) and solvent, as well as a catalyst solution containing the HPA-5 catalyst (*c*_catalyst_ = 12 mmol/L) and solvent, were prepared. The solvent was either 100% deionized water (aqueous system) or a mixture of 90:10 vol % H_2_O/Methanol (aqueous-methanolic system). The prepared solutions were filled into their corresponding liquid containers and pressurized with nitrogen to approximately 120% of the desired system pressure (around 30 bar). To ensure that both the reactors and the sampling loop did not contain residues of previous experiments, the system was purged with nitrogen for at least 10 min. Afterward, the sample loop was flushed with the reaction solution to ensure that no water from prerinsing the system is diluting the corresponding samples. To remove nitrogen and remaining liquid from previous experiments, the gas supply line was thoroughly flushed with oxygen for a minimum of 15 min. The sample loop was then submerged in a Dewar filled with water and crushed ice (0–10 °C) to condense as much of the reaction mixture as possible during sample taking. The samples were then prepared for analytics.

### Catalytic Experiments in the Stirred-Tank Reactor (STR)

To evaluate the performance of the Taylor-flow microreactor setup in xylose conversion, comparative tests were conducted in a classical stirred-tank reactor (STR) under identical reaction conditions. Therefore, a 3-fold high-pressure oxidation plant equipped with gas entrainment stirrers and 100 mL batch autoclaves was used (Figure S2). All autoclaves, pipes, valves, and fittings were made of stainless steel 1.4571. The gaskets were made of Novaphit MST/XP supplied by Erwin Telle GmbH. In a typical experiment, glass liners were filled with 12 mmol/L HPA-5 catalyst, 50 mmol/L xylose as a substrate, and 45 mL of solvent. Here, either 100% water or a mixture of 90:10 vol % H_2_O/MeOH were used. The filled glass liners were installed in the autoclaves. Afterward, the autoclaves were purged three times with 35 bar of oxygen. Before the start of the experiment, the autoclaves were pressurized with oxygen to 43 bar. Subsequently, the reaction temperature and stirrer speed were set to 100 °C and 300 rpm. After the temperature was reached, the zero-min sample was drawn from the sampling valve. The stirrer speed was subsequently increased to 1000 rpm. Samples were drawn after the corresponding residence times of the microreactor setup. When the reaction was finished, the stirrer speed was decreased to 300 rpm, the heating jackets were taken off, and the reactors were cooled with pressurized air. After cooling the reactors to room temperature, samples of the gas phase were taken, and subsequently, the autoclaves were vented. Afterward, the samples were prepared for NMR spectroscopy and HPLC as well as GC-TCD measurements.

### Calculation of Performance Indicators

For continuous microreactor reactions, all products were quantitatively analyzed using high-performance liquid chromatography (HPLC) and Gas Chromatography (GC). The concentrations of xylose and of all reaction intermediates were determined using an Agilent 1260 Infinity II HPLC equipped with an Aminex HPX-87H Organic Acid column and a refractive index detector (RID). For the analysis of volatile substances, GC was utilized. Here, an Agilent 8890 GC system equipped with a CP-Sil 5 CB column and a flame ionization detector (FID), together with a methanizer from Polyarc and a thermal conductivity detector (TCD) was used. Xylose conversion *X*_xylose_ was calculated using eq 5, where ṅ represents the corresponding molar flows

5 $$X_{\mathrm{xylose}}=\frac{{ṅ}_{\mathrm{xylose},\mathrm{in}}-{ṅ}_{\mathrm{xylose},\mathrm{out}}}{{ṅ}_{\mathrm{xylose},\mathrm{in}}}=1-\frac{{ṅ}_{\mathrm{xylose},\mathrm{out}}}{{ṅ}_{\mathrm{xylose},\mathrm{in}}}$$

Molar flows were calculated from liquid concentrations *c_i_* and liquid volumetric flow rates *V̇*. Yields *Y*_*i*_ were calculated using eq 6, taking stoichiometric coefficients *v*_*i*_ into account.

6 $$Y_{i}=\frac{{ṅ}_{i,\mathrm{out}}-{ṅ}_{i,\mathrm{in}}}{{ṅ}_{\mathrm{xylose},\mathrm{in}}}\frac{\left|v_{\mathrm{xylose}}\right|}{v_{i}}$$

Evaluation of the results from the STR studies was performed as follows: Liquid phase analysis was carried out using a Nexera-40 HPLC from Shimadzu equipped with a polymer-based 300 mm × 8.0 mm Organic Acid column by CS-Chromatographie GmbH and a refractive index detector. Four mmol of an aqueous sulfuric acid solution was used as eluent, and the samples were filtered through a syringe filter (45 μm) before analysis. The yields of formic acid and methyl formate were quantified by ^1^H NMR using a Bruker Avance III HD 600 MHz spectrometer. Yields were calculated as *n*(product)/*n*(#C atoms xylose). The determination of the gaseous byproducts CO_2_ and CO was done by means of GC analysis using a Varian GC 450 equipped with a 2 m × 0.75 mm ID ShinCarbon ST column and a TCD detector. Yields were calculated as *n*(CO_2_ versus CO)/*n*(C atoms substrate). No other gaseous products could be detected by the used GC.

## Supplementary Material



## Data Availability

Research data are available/will be made available after official publication (10.5281/zenodo.14916673).

## Notes

Corresponding P&I flowcharts of used reactor setups; IR spectrum of catalyst, NMR spectra of reaction solutions and UV–vis spectra of catalyst solutions; experimental setup for the investigation of hydrodynamics, oxygen solubility, and heat transfer measurements; mass transfer coefficient values; reproduction experiments; further results for variation of reaction parameters (pressure variation, gas hold-up variation, two-phase velocity variation); and testing of Renmatix (PDF)
