# Supplementary material for: Comparing the Polyoxometalate-Catalyzed Oxidation of C5-Containing Biomass to Formic Acid in a Taylor-Flow Microreactor and a Stirred-Tank Reactor
Source: ACS Sustain Chem Eng. 2025 Jul 24;13(30):11999–2009. doi: 10.1021/acssuschemeng.5c03392 (PMC12326389; doi:10.1021/acssuschemeng.5c03392)
Supplement: Supplementary file 1 [file sc5c03392_si_001.pdf]

Supporting Information for

Comparing the polyoxometalate-catalyzed oxidation of C5-containing biomass to formic acid in a Taylor-flow microreactor and a stirred tank reactor

Jan-Dominik H. Krueger[1], Lukas Popp[2], Markus Schörner[3], Hans Lorenz Grau[2], Patrick Schühle[*2], Jakob Albert*[*1]*

[1] Institute of Technical and Macromolecular Chemistry, Universität Hamburg, Bundesstrasse 45, 20146 Hamburg, Germany

[2] Institute of Chemical Reaction Engineering, Friedrich-Alexander-Universität Erlangen-Nürnberg, Egerlandstr. 3, 91058 Erlangen, Germany

[3] Forschungszentrum Jülich, Helmholtz Institute Erlangen Nürnberg for Renewable Energy, 91058 Erlangen, Germany

Contents

Set-up of Taylor-flow microreactor device: 2

Set-up of stirrer-tank reactor device: 2

Catalyst analysis: 3

Investigation of hydrodynamics and oxygen solubility in the Taylor flow microreactor setup: 4

Xylose oxidation in the microreactor under standard reaction conditions 6

Pressure variation in Taylor flow reactor down to 1.0 bar O_2 8

Variation of gas hold-up in Taylor flow reactor 9

Variation of two-phase velocity in Taylor flow reactor 9

Experiments with Renmatix as exemplary C5-biomass 10

References 10

This supporting information contains 18 figures and 2 tables on 10 pages.

Set-up of Taylor-flow microreactor device:

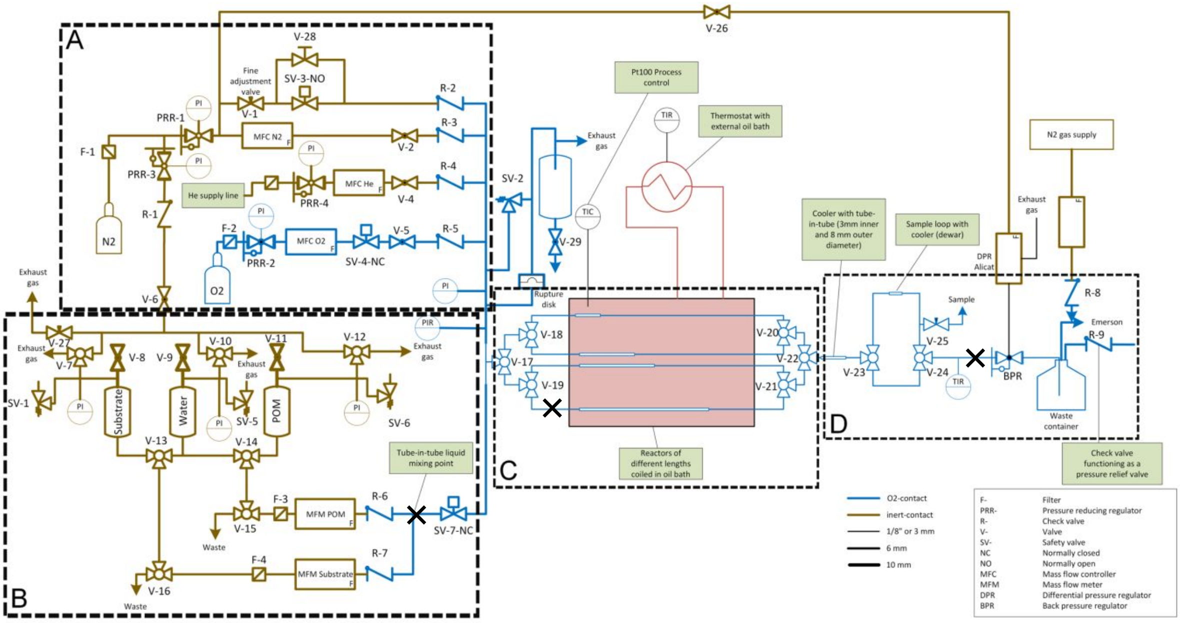

Figure S1: P&I flow chart of used microreactor setup. A) Gas supply lines, B) Liquid supply, C) Tubular reactors in oil bath, D) Sample loop with downstream waste container. Measurement points for analysis of Taylor flow formation are marked by black crosses.

Set-up of stirrer-tank reactor device:

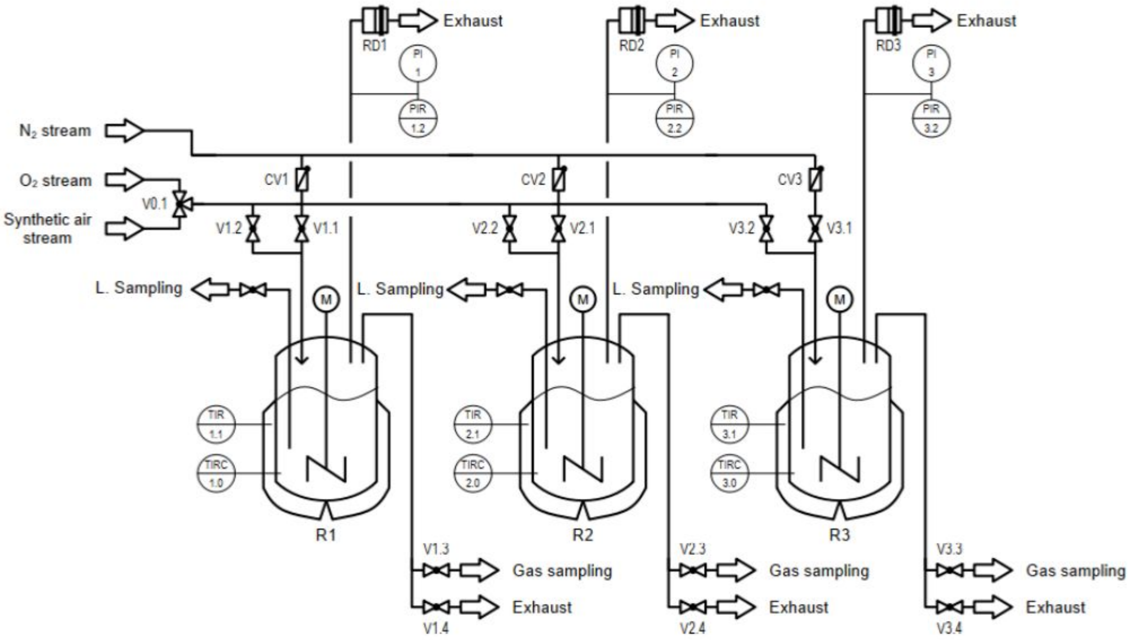

Figure S2: P&I flow chart of used stirred tank reactor setup.

Catalyst analysis:

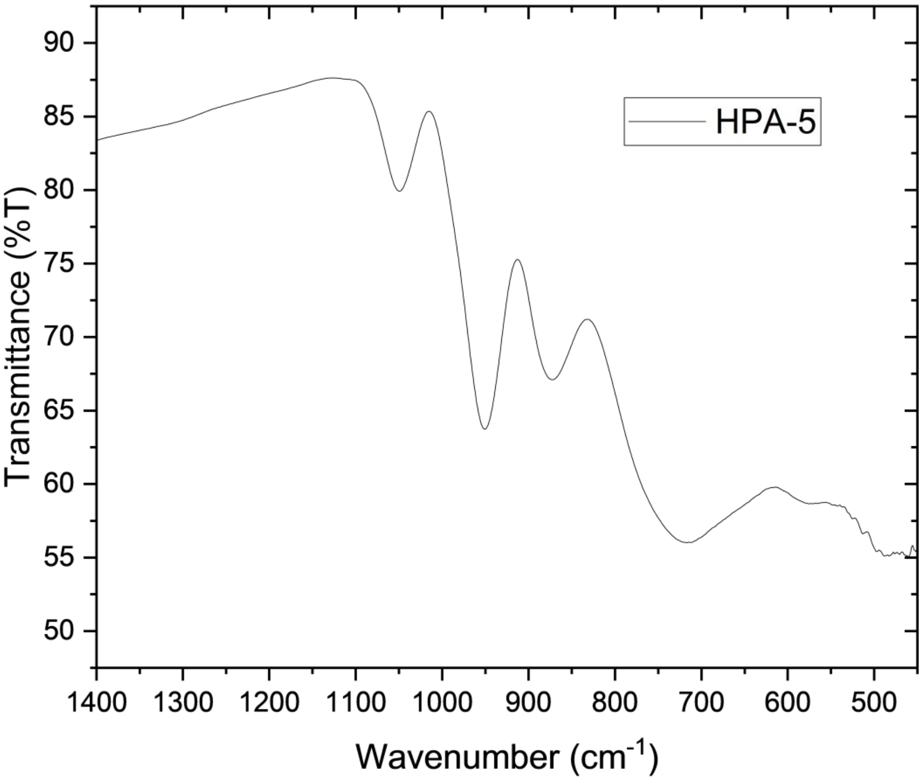

Figure S3: FT-IR spectrum of used HPA-5 catalyst measured with an IRSpirit-X IR spectrometer equipped with an ATR unit from Shimadzu.

Investigation of hydrodynamics and oxygen solubility in the Taylor flow microreactor setup:

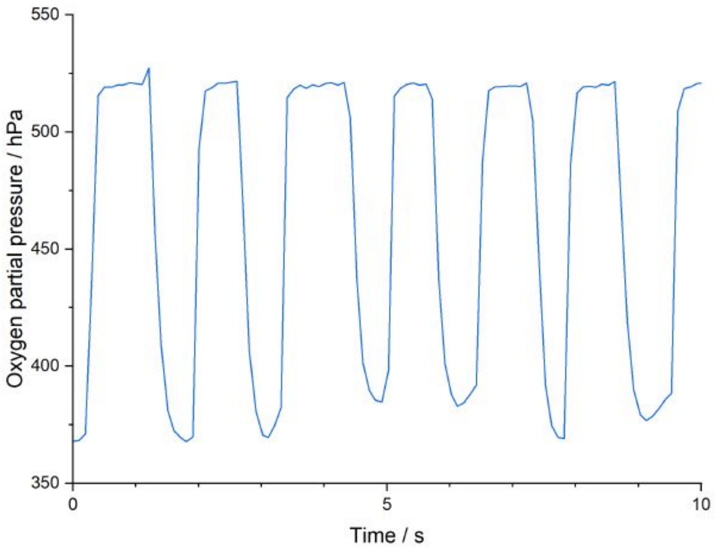

Figure S4: Oxygen concentration during the Taylor flow at p = 9 bar, p_{O2} = 0.525 bar, T = 21 ◦C, $\dot{V}_{N2}$ = 6.5 mL^N min^{-1}, $\dot{V}SA$ = 2.5 mL^N min^{-1}, $\dot{V}_{H2O}$ = 1 mL min^{-1}, ε_G = 0.5.

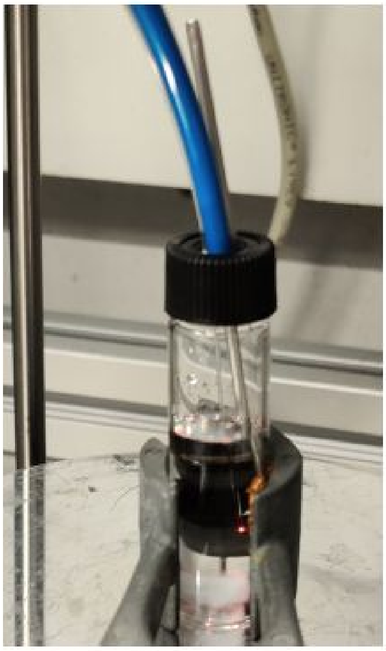

Figure S5: Setup for liquid reference sample oxygen measurement.

Table S1: Liquid slug and bubble lengths for various gas hold-ups ε_G. Measurement conditions: water, nitrogen, d_c = 2 mm, p_a = 10 bar, T = 21 °C, V_G = 0.667 – 4 mLmin^{-1}.

**Parameters / gas hold-up ε_G**	**L_{UC} / cm**	**L_b / cm**	**L_{slug} / cm**
**0.25**	2.5	0.7	1.8
**0.333**	2	0.7	1.3
**0.5**	1.6	0.8	0.8
**0.667**	1.6	1.1	0.5
**0.75**	2.1	1.6	0.5

Table S2: Properties of water and other parameters for the calculation of k_La from empirical correlations.[1,2]

**d_c / m**	**$D_{O2,L}$ / m^2s^{-1}**	**ρ_L / kg m^{-3}**	**μ_L / P a s**	**U_{TP} / m s^{-1}**
0.002	$2 \cdot 10^{-9}$	998.2	0.001	0.01061

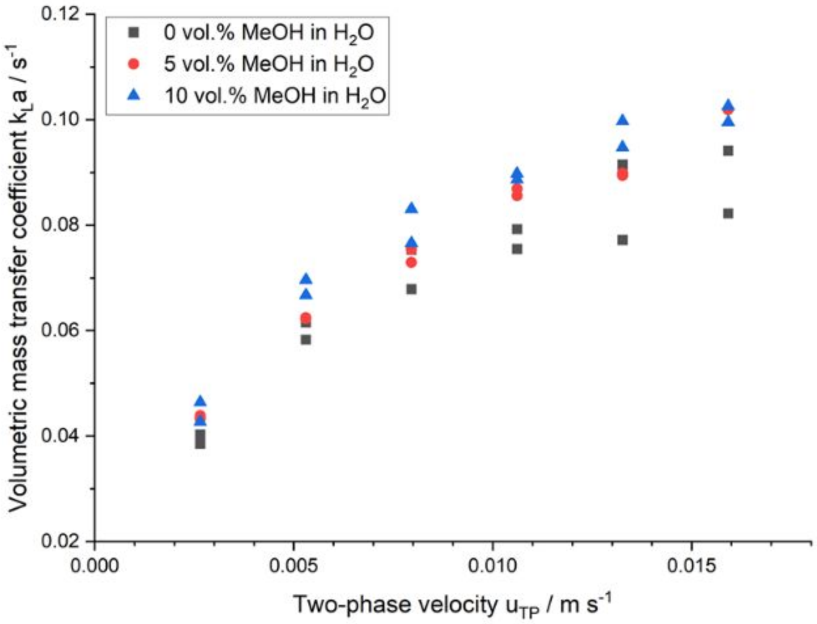

Figure S6: Mass transfer coefficient k_La versus two-phase velocity u_{TP} in methanolic solution. Experimental values (each setpoint measured twice). Measurement conditions: solvent as indicated, p_{total} = 9bar, p_{O2} = 0.525 bar, ε_G = 0.5, l_{tube} = 12 cm. Gas hold-up was kept constant while the total volumetric flow rate was adjusted.

Xylose oxidation in the microreactor under standard reaction conditions

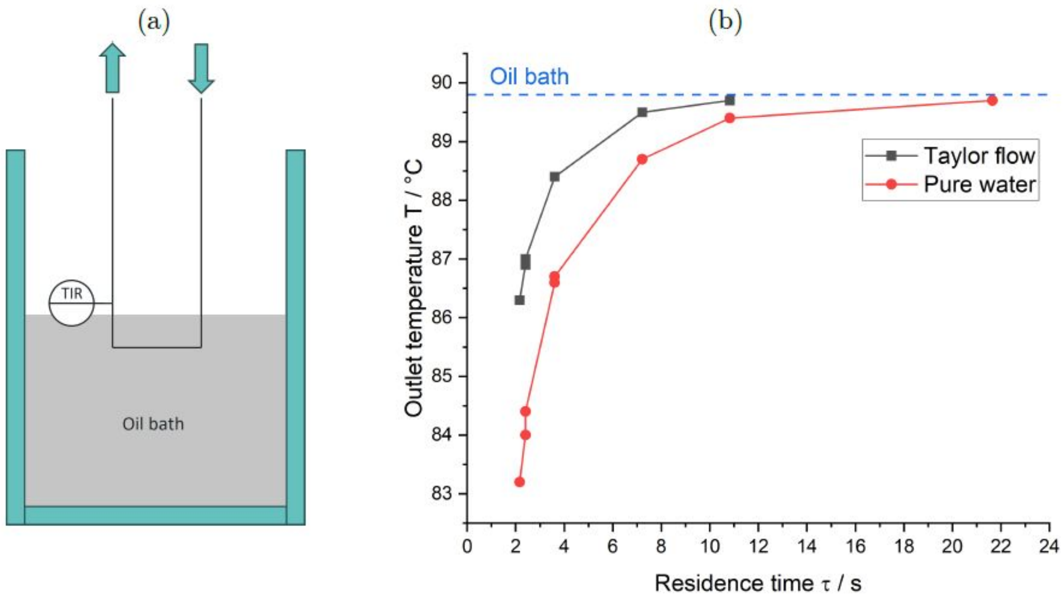

Figure S7: Scheme of measurement setup and outlet temperature for varied residence time to see heat transfer.

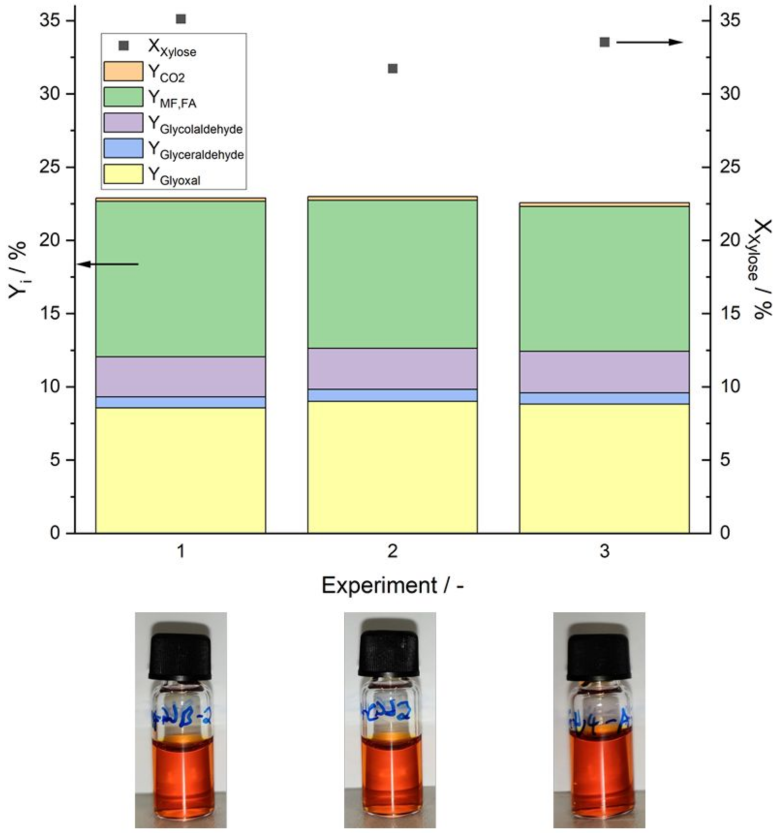

Figure S8: Threefold reproduction of the same experiment in methanolic solution. Conversion of xylose and yield of intermediates and products. Reaction conditions: c_{xylose} = 50mmolL^{-1}, cHPA−5 = 12mmolL^{-1}, φ_{MeOH} = 10 vol.%, p_{total} = 25 bar, p_{O2} = 25 bar, T = 100 °C, ε_G = 0.5, τ = 10min. Yields of methyl formate and formic acid are combined. Below are images of the sample vials with the respective liquid sample.

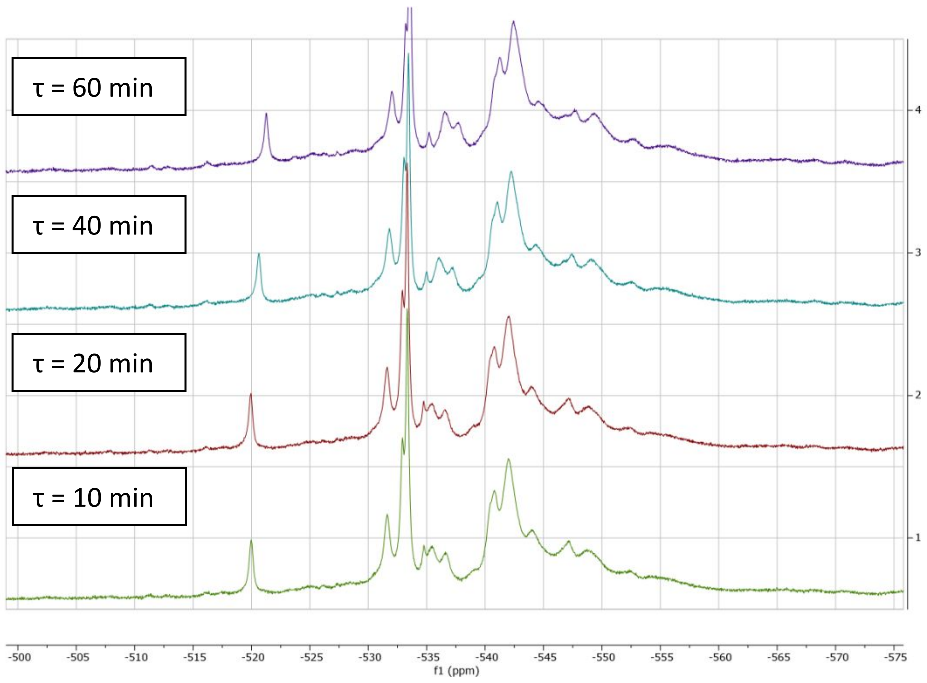

Figure S9: ^{51}V-NMR spectra of aqueous reaction solutions for residence time variation.

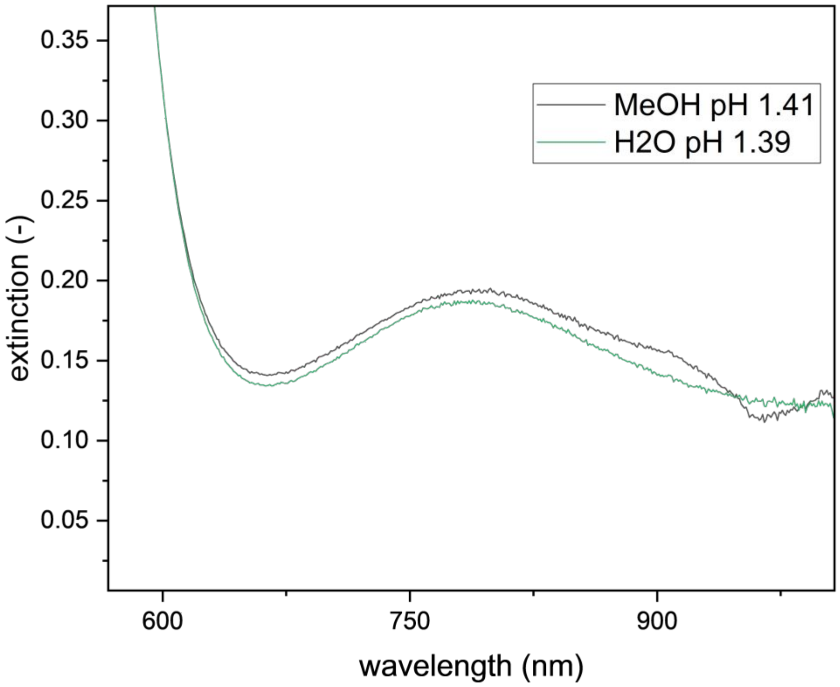

Figure S10: Corresponding UV-Vis spectra for dissolved catalyst in different solvent mixtures.

Pressure variation in Taylor flow reactor down to 1.0 bar O_2

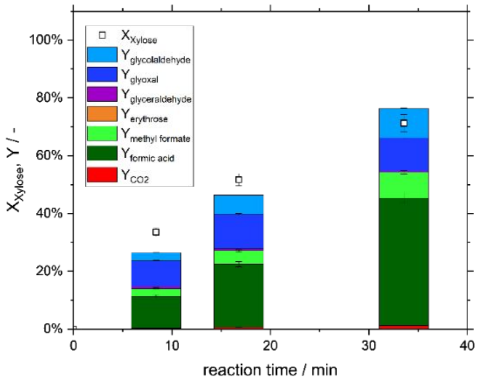

Figure S11: Conversion and yields for methanolic system at 25 bar O_2 pressure.

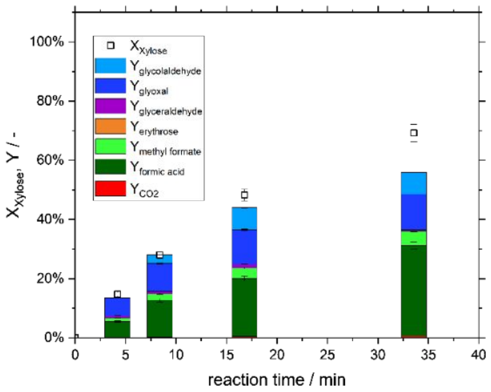

Figure S12: Conversion and yields for methanolic system at 10 bar O_2 pressure

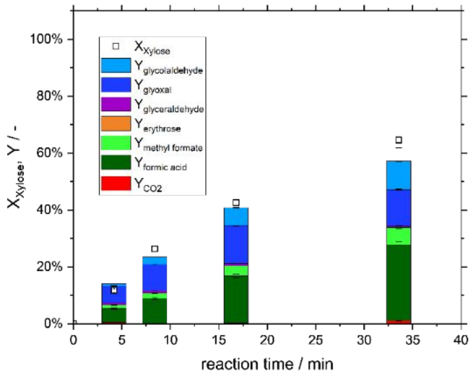

Figure S13: Conversion and yields for methanolic system at 5 bar partial O_2 pressure

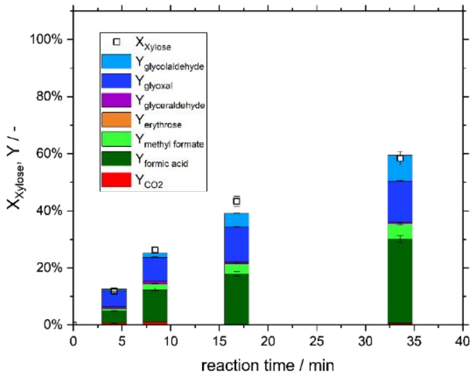

Figure S14: Conversion and yields for methanolic system at 2.5 bar partial O_2 pressure

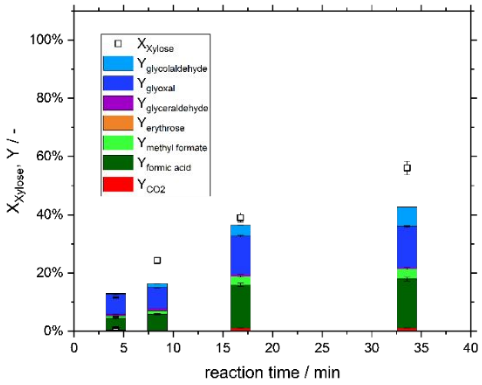

Figure S15: Conversion and yields for methanolic system at 1 bar partial O_2 pressure

Variation of gas hold-up in Taylor flow reactor

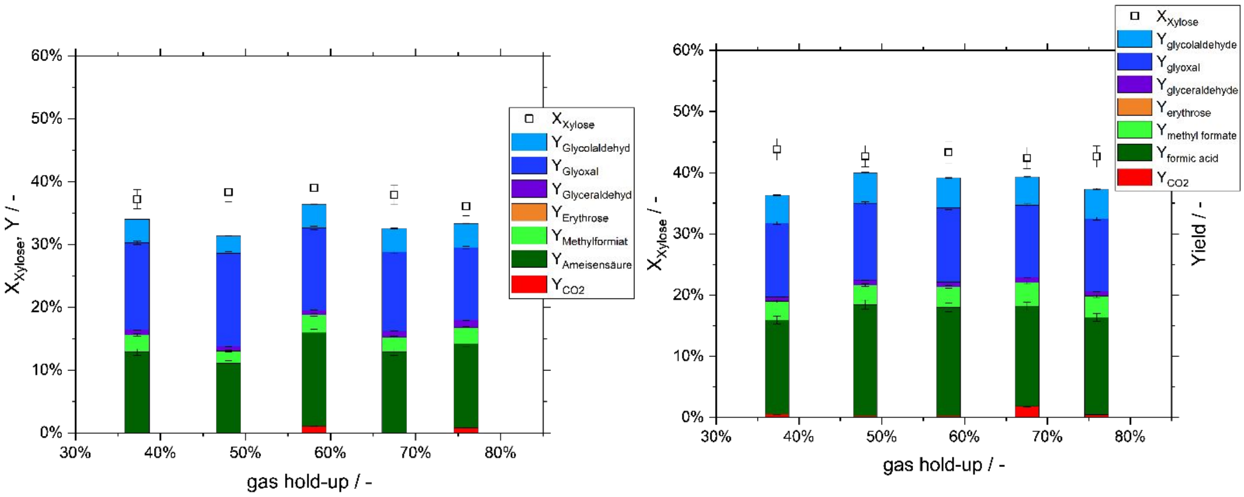

Figure S16: Variation of gas hold-up at 1 bar (left) and 2.5 bar (right) partial oxygen pressure.

Variation of two-phase velocity in Taylor flow reactor

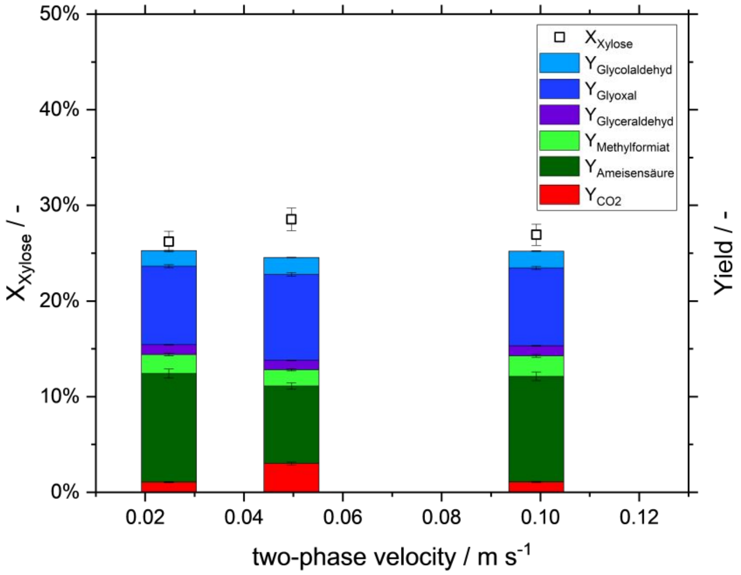

Figure S17: Variation of two-phase velocity for 2.5 bar oxygen pressure. Reaction conditions: c(Xylose) = 50 mmol/L, c(HPA-5) = 12 mmol/L, T = 100 °C, solvent composition: 90:10 vol.% H_2O:MeOH; τ = 8.4 min. ε_G = 0.58.

Experiments with Renmatix as exemplary C5-biomass

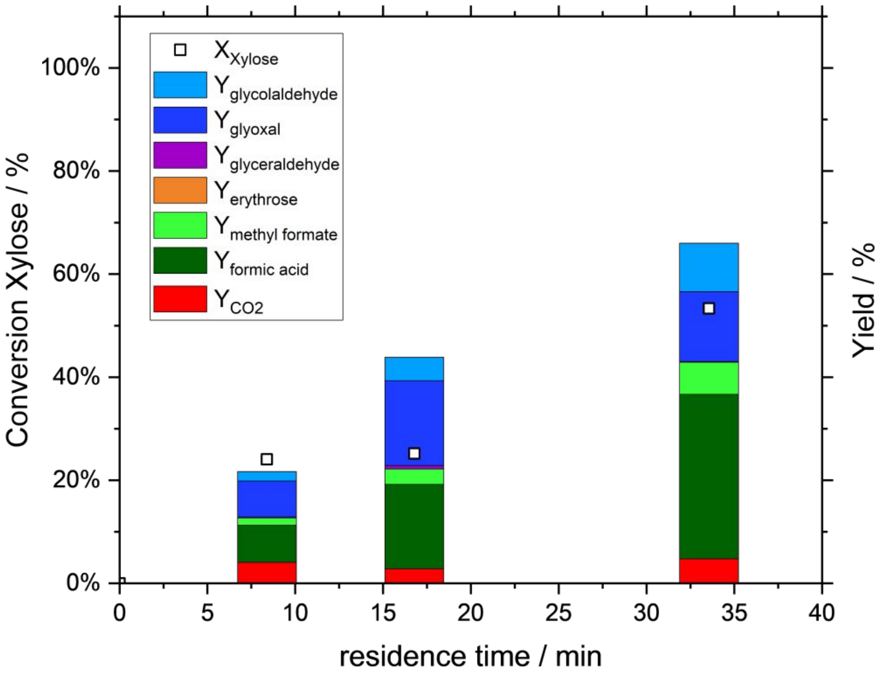

Figure S18: Residence time variation for real biomass substrate Renmatix in methanolic solution at 2.5 bar partial oxygen pressure.

References

(1) Numerical Data and Functional Relationships in Science and Technology. Subvol. A: Group 4, Physical Chemistry Bd. 15. Diffusion in Gases, Liquids and Electrolytes Gases in Gases, Liquids and Their Mixtures, Winkelmann, J., Ed.; Springer: Berlin Heidelberg New York, 2007.

(2) Kashid, M. N.; Renken, A.; Kiwi-Minsker, L. *Microstructured Devices for Chemical Processing*; Wiley-VCH: Weinheim, 2015.
